# Supplementary figures and images for: Salmonella Enteritidis Effector AvrA Suppresses Autophagy by Reducing Beclin-1 Protein
Source: Front Immunol. 2020 Apr 17;11:686. doi: 10.3389/fimmu.2020.00686 (PMC7181453; doi:10.3389/fimmu.2020.00686)

Fig. S1

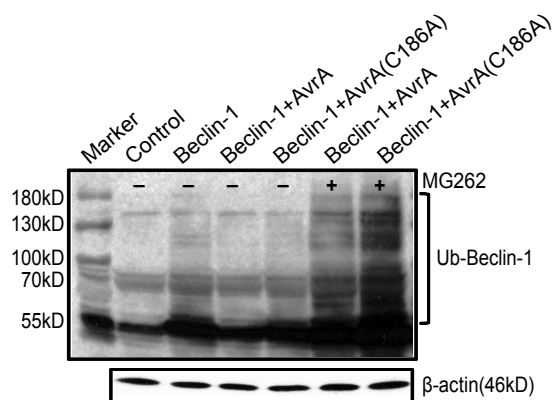

Supplement: Figure S1 — AvrA decrease the ubiquitination of Beclin-1. The HCT116 cells were transfected with the indicated plasmids (200 ng/μl, 24 h incubation, n = 3) and were incubated for 2 h with the proteasome inhibitor MG262 (40 μmol/L). The total cell lysates were analyzed for ubiquitinated Beclin-1 by an immunoblot. The higher-molecular weight ubiquitinated Beclin-1 is indicated by bracket. The data shown are from a single experiment and are representative of three separate experiments. [file Image_1.pdf]
